# *GNAS1*基因T393C多态性与*EGFR*突变状态未明复治晚期非小细胞肺癌TKI疗效的关联研究

**DOI:** 10.3779/j.issn.1009-3419.2014.04.06

**Published:** 2014-04-20

**Authors:** 卫 洪, 宝钗 林, 贝贝 张, 伟敏 毛, 沂平 张

**Affiliations:** 1 310022 杭州，浙江省肿瘤医院，浙江省胸部肿瘤诊治技术研究重点实验室 Zhejiang Cancer Hospital, Zhejiang Key Laboratory of the Diagnosis and Treatment Technology on Thoracic Oncology, Hangzhou 310022, China; 2 325000 温州，温州医学院附属第一医院肿瘤内科 Department of Medical Oncology, the First Affiliated Hospital of WenZhou Medical College, Wenzhou 325000, China; 3 310053 杭州，浙江中医药大学第二临床医学院 The Second Clinical Medical College, Zhejiang Chinese Medical University, Hangzhou 310053, China

**Keywords:** *GNAS1*基因, 多态性, 肺肿瘤, 酪氨酸激酶抑制剂, *GNAS1* Gene, Polymorphism, Lung neoplasms, Tyrosine kinase inhibitor

## Abstract

**背景与目的:**

晚期非小细胞肺癌（non-small cell lung cancer, NSCLC）肿瘤组织表皮生长因子受体（epidermal growth factor recetor, *EGFR*）突变是酪氨酸激酶抑制剂（tyrosine kinase inhibitor, TKI）最重要的疗效预测指标，但患者常常因肿瘤组织量太少导致*EGFR*突变状态未明。TKI可以诱导肿瘤细胞凋亡，并与许多凋亡相关基因表达相关。通过检测*GNAS1*基因T393C多态性，探讨其与*EGFR*突变状态未明的复治晚期NSCLC小分子TKI治疗疗效的关系。

**方法:**

入组2009年1月1日-2012年4月30日就诊于浙江省肿瘤医院的116例复治晚期NSCLC患者，所有患者既往均接受过化疗，进展后接受吉非替尼或厄洛替尼靶向治疗。采用多聚酶链反应方法检测患者外周血白细胞中*GNAS1*基因T393C多态性。采用SPSS 18.0统计软件分析。

**结果:**

总有效率29.3%，*GNAS1*基因T393C各基因型患者间的有效率无明显差异。相比*GNAS1*基因其它基因型，CC型疾病控制率更低（46.2% *vs* 73.8%, *P*=0.039）。单因素分析中位PFS，CC型中位无进展时间短于其它基因型（2.3个月*vs* 6.0个月，*P*=0.005），而女性长于男性（10.2个月*vs* 4.6个月，*P*=0.04)；不吸烟者长于有吸烟史者（11.9个月*vs* 2.5个月，*P* < 0.001)；病理类型为腺癌长于其他类型（11.9个月*vs* 4.1个月，*P* < 0.001)，均达到统计学差异。多因素分析结果显示，包括吸烟史、ECOG评分和病理类型、*GNAS1*基因多态性为PFS的独立预后因素（*P*=0.006）。

**结论:**

对复治晚期*EGFR*突变状态未明的NSCLC，*GNAS1*基因T393C基因型为CC者是提示近期疗效较差的指标。

## Abstract (en)

**Background and objective:**

Epidermal growth factor receptor (EGFR)-activating mutation predicts excellent response to EGFR tyrosine kinase inhibitors (TKIs). However, lung cancer patients are often with unknown *EGFR* mutation status because there are little tumor specimen to determine. TKIs induce tumor cell apoptosis which associates with several apoptosis-related genes. To explore the association between *GNAS1* T393C polymorphism and therapeutic efficacy of TKI in pretreated advanced non-small cell lung cancer (NCSLC) with unknown *EGFR* mutation status.

**Methods:**

A total of 116 patients were recruited for the study from Zhejiang Cancer Hospital, all of whom were treated with gefitinib or erlotinib after failure to prior chemotherapy. We detected the genotype of peripheral blood lymphocytes of patients with *GNAS1* T393C polymorphism through polymerase chain reaction (PCR). Statistical analysis was performed by SPSS version 18.0.

**Results:**

The overall response rate was 29.3%. No significant associations were found among *GNAS1* T393C polymorphism and the objective response rate. The disease control rate of patients with *GNAS1* T393C CC genotype was lower than that of patients with variant genotype (TT or CT) (46.2% *vs* 73.8%, *P*=0.039). Univariate analysis identified gender, smoking history, histology and *GNAS1* T393C polymorphism as predictive marker of PFS (*P*=0.04, *P* < 0.001, *P* < 0.001 and *P*=0.005). Multivariate analysis of factors, including smoking history, performance status score, histology, *GNAS1* T393C polymorphism demonstrated that *GNAS1* T393C polymorphism was correlated independently with PFS (*P*=0.007).

**Conclusion:**

Our data suggest the role of *GNAS1* T393C CC genotype as a poor predictive marker both of DCR and PFS in advanced NSCLC patients treated with tyrosine kinase inhibitor.

晚期非小细胞肺癌（non-small cell lung cancer, NSCLC）患者肿瘤组织表皮生长因子受体（epidermal growth factor recetor, *EGFR*）突变是酪氨酸激酶抑制剂（tyrosine kinase inhibitor, TKI）最重要的疗效预测指标^[1]^，但常常因各种原因导致的肿瘤组织量太少以致*EGFR*突变状态未明，ISEL研究的数据中能够获得足量肿瘤组织标本进行*EGFR*突变检测标本的患者仅约20%^[2]^。BR21^[3]^、TITAN^[4]^和INTEREST^[5]^等临床研究显示了厄洛替尼和吉非替尼在晚期*EGFR*突变未明NSCLC患者二、三线治疗中的疗效。目前NCCN指南指出在二线及二线以上治疗时，对*EGFR*突变状态未明的患者可以选择TKI，如何对这些患者进行治疗前的疗效预估是我们面临的临床难题。研究^[6]^发现，TKI的一个重要作用机制是诱导肿瘤细胞凋亡。*GNAS1*基因位于20号染色体，包含13个外显子，其中T393C多态性与肿瘤组织Gs蛋白α亚单位mRNA表达增加相关^[7]^。而且，Gs蛋白α亚单位表达增加会促进凋亡发生^[8]^。本研究探索晚期NSCLC患者*EGFR*突变未明TKI近期疗效与促凋亡基因*GNAS1*基因T393C多态性的关系，报告如下。

## 1 资料和方法

### 1.1 研究对象

浙江省肿瘤医院化疗中心2009年1月-2012年4月间收治的116例晚期NSCLC患者，经组织学或细胞学确诊的NSCLC，具有可测量病灶，肝肾功能不超过1度异常，既往经过一至二线化疗后病情进展，各种原因导致无法明确患者肿瘤组织*EGFR*突变状态，均可考虑入组接受二线或三线EGFR-TKI靶向治疗。排除标准为：既往患间质性肺病、药物诱导的间质性疾病、需要激素治疗的放射性肺炎或任何具临床证据的活动性间质性肺病；基线时CT扫描发现存在特发性肺纤维化；没有完全控制的眼部炎症或眼部感染，或任何可能导致上述眼部疾病的情况；既往有明确的神经或精神障碍史，包括癫痫或痴呆；混有小细胞肺癌成分的患者。

所有研究对象采血前均对本研究知情同意并签署知情同意书，本研究获得浙江省肿瘤医院医学伦理委员会审核批准。

### 1.2 方法

抽取患者治疗前外周静脉全血2 mL，EDTA抗凝，采用全血基因组DNA提取试剂盒（康为世纪公司），按照说明书进行基因组DNA提取，提取后-80 ℃冰箱保存备用。用紫外分光光度计测定DNA浓度和纯度。OD_260_/OD_280_比值的参考值为1.8-2.0。

#### 1.2.1 PCR扩增

引物序列为：上游引物5′-CTCCTAACTGACATGGTGCAA-3′，下游引物5′- TAAGGCCACACAAGTCGGGGT-3′。PCR反应总体积为25 μL，2^*^GoldStar Taqman Mixture（with ROX）12.5 μL，上、下游引物各1 μL，样本2 μL，去离子水8.5 μL。PCR的反应条件为：95 ℃×10 min预变性；95 ℃×30 s变性，60 ℃×30 s退火，72 ℃×60 s延伸，72 ℃×5 min终延伸，35个循环。本实验已设空白对照孔，每个样本均设置复孔。

#### 1.2.2 琼脂糖凝胶电泳体系

将TAE缓冲液、1%琼脂糖、溴化乙锭（0.5 μg/mL）在电压120 V的琼脂糖凝胶电泳体系电泳60 min。

### 1.3 疗效评价标准

疗效评定采用实体瘤疗效评价标准（Response Evaluation Criteria in Solid Tumors, RECIST 1.1）^[9]^，分为完全缓解（complete response, CR）、部分缓解（partial response, PR）、稳定（stable disease, SD）和进展（progressive disease, PD）。客观缓解率（objective response rate, ORR）=（CR+PR）/（CR+PR+SD+PD）×100%。疾病控制率（disease control rate, DCR）=（CR+PR+SD）/（CR+PR+SD+PD）×100%。

### 1.4 随访和生存分析

随访采用门诊、电话或书信方式，末次随访时间为2012年11月30日。无进展生存期（progression-free survival, PFS）定义为患者自接受吉非替尼或厄洛替尼靶向治疗开始至疾病进展、死亡或不良反应不可耐受。对于在随访截止日期无进展的病例，在统计时作截尾数据处理。

### 1.5 统计学方法

采用SPSS 18.0统计软件，分析基因多态性与疗效评价的关系采用χ^2^检验，单因素分析PFS采用*Kaplan-Meier*生存曲线分析及*Log-rank*检验，多因素分析采用*Cox*分析法，以*P* < 0.05表示差异有统计学意义。

## 2 结果

### 2.1 一般临床特征

入组患者共116例，其中共41例患者服用吉非替尼（易瑞沙）250 mg，每天1次口服治疗；75例患者服用厄洛替尼（特罗凯）150 mg，每天1次口服治疗。所有患者具体临床特征详见（表 1）。

**1:** 患者的一般临床特征 General clinical characteristics of the patients

Variable	*n*	Proportion (%)
Age (year)		
≥60	37	31.9
< 60	79	68.1
Gender		
Male	61	52.6
Female	55	47.4
Smoking history		
Yes	43	37.1
No	73	62.9
Performance status score		
0-1	90	77.6
2	26	22.4
Pathological type		
Adenocarcinoma	87	75.0
Other	29	25.0
Disease stage		
Ⅲ	21	18.1
Ⅳ	95	81.9
Targeted drug		
Gefitinib	41	35.3
Erlotinib	75	64.7
Line		
Second line	70	60.3
Third line	46	39.7

### 2.2 *GNAS1*基因多态性检测结果判定

扩增出的目的基因为*GNAS1*基因的一个片段，长度为345 bp，这个片段中可能含有限制性内切酶*Fok*I的酶切识别位点：5′-CGATG(N)9↓-3′，3′-CCTAC(N)13↑-5′。用*FOK*I酶切*GNAS1*基因片段：将该反应体系放入37 ℃恒温水浴箱中5 h后，用1.5%的琼脂糖凝胶150 V电泳30 min，观察酶切图谱。有些PCR产物的核苷酸链中的ATT转换为ATC，可被FOKI识别而切为259 bp和86 bp两段。条带被完全切开后的259 bp，即为*GNAS1*基因的CC型；条带未被酶切的345 bp片段，为TT型；条带为双链DNA片段一条被酶切，一条未切开的产物酶谱，为*GNAS1*基因TC型（图 1）。

**1:**
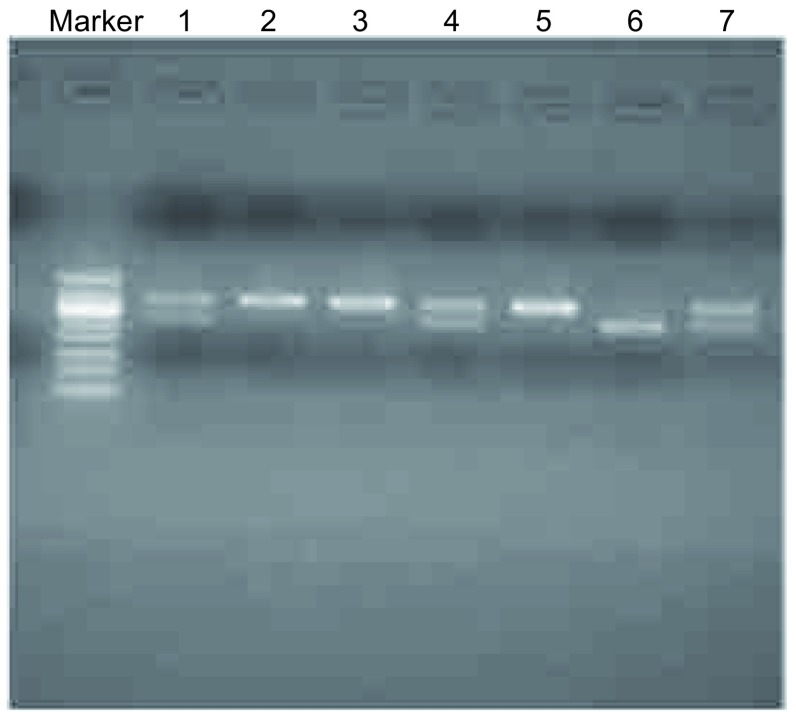
*GNAS1*基因T393C多态性判定图。在凝胶成像系统扫描中最左侧为maker电泳通道，*GNAS1*基因的基因型为第1、4、7通道为TC型，第2、3、5为CC型，第6通道为TT型 *GNAS1* T393C gene polymorphism detection result diagram. The leftmost of the gel imaging system is maker electrophoresis channel, the 1, 4 and 7 passage is TC genotype, channel 2, 3 and 5 represents CC genotype, channel 6 is TT genotype

### 2.3 *GNAS1*基因片段多态性与TKI治疗ORR的关系

所有患者共116例，总有效率29.3%，其中*GNAS1*基因T393C基因型为TT型、CT型及CC型分别为46例、57例和13例，有效率分别为38.5%、33.3%和21.7%，*GNAS1*基因各基因型患者间的有效率无明显差异。相比*GNAS1*基因其它基因型，CC型疾病控制率更低（46.2% *vs* 73.8%, *P*=0.039）（表 2）。

**2:** *GNAS1*基因T393C基因多态性与TKI治疗疗效的关系 Relationship between *GNAS1* T393C gene polymorphism and therapeutic efficacy of tyrosine kinase inhibitor (TKI)

Genotype	Therapeutic evaluation		*χ*^2^	*P*	Therapeutic evaluation		*χ*^2^	*P*
	CR+RR (*n*)	SD+PD (*n*)			CR+RR+SD (*n*)	PD (*n*)		
TT	11	35	1.11	0.58	30	16	7.2	0.027
CT	19	38			46	11		
CC	4	9			6	7		
TT+CT	30	73	0.015	0.90	76	27	4.25	0.039
CR: complete response; PR: partial response; SD: stable disease; PD: progressive disease.								

### 2.4 单因素分析PFS

单因素分析中位PFS，*GNAS1*基因型为CC型中位PFS短于其它基因型（2.3个月*vs* 6.0个月，*P*=0.005），而女性长于男性（10.2个月*vs* 4.6个月，*P*=0.04）；不吸烟者长于有吸烟史者（11.9个月*vs* 2.5个月，*P* < 0.001）；病理类型为腺癌长于其他类型（11.9个月*vs* 4.1个月，*P* < 0.001），均存在统计学差异，见表 3和图 2。

**3:** 单因素分析无进展生存时间 Univariate analysis of progression-free survival (PFS)

Variable	PFS (median)	*χ*^2^	*P*
Age (year)		0.005	0.94
≥60	5.0		
< 60	4.9		
Gender		4.24	0.04
Male	4.6		
Female	10.2		
Smoking history		15.28	< 0.001
Yes	2.5		
No	11.9		
Performance status score		0.95	0.35
0-1	4.8		
2	5.0		
Pathological type		13.06	< 0.001
Adenocarcinoma	11.9		
Other	4.1		
Disease stage		1.05	0.31
Ⅲ	4.7		
Ⅴ	5.0		
Targeted drug		0.75	0.39
Gefitinib	5.0		
Erlotinib	5.0		
Line		1.22	0.27
Second line	5.0		
Third line	4.7		
*GNAS1* genotype		7.77	0.005
TT+CT	6.0		
CC	2.3		

**2:**
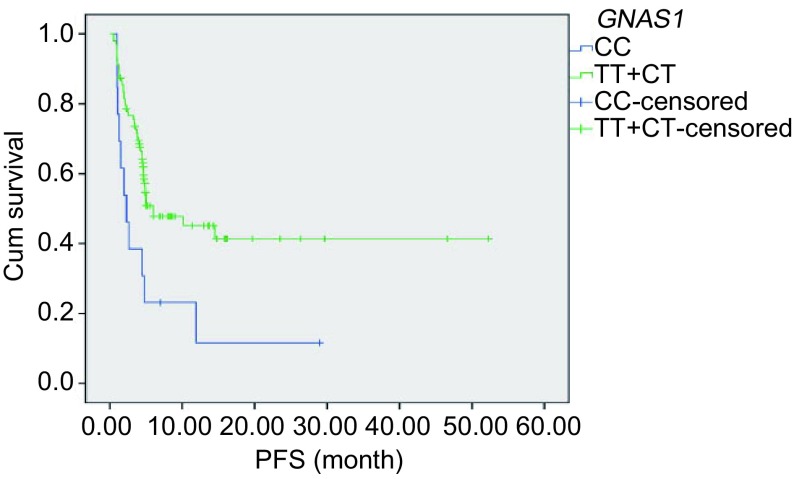
单因素分析*GNAS1*基因T393C多态性与PFS的关系 Univariate analysis of the relationship between PFS and *GNAS1* T393C gene polymorphism

### 2.5 多因素分析PFS

多因素分析结果显示，包括PS评分、吸烟史和病理类型在内，*GNAS1*基因T393C多态性为PFS的独立预后因素（*P*=0.006），见表 4。

**4:** 多因素分析PFS Multivariate analysis of PFS

Variable	Wald	Sig.	Exp(B)	95%CI for Exp(B)
Age	0.002	0.97	0.987	0.544-1. 790
Gender	0.731	0.393	1.481	0.6028-3.642
Smoking history	9.159	0.002	0.264	0.111-0.625
Performance status score	3.797	0.050	0.482	0.231-1.004
Pathological type	10.968	0.001	2.811	1.525-5.182
Disease stage	0.948	0.330	1.419	0.702-2.868
Targeted drug	0.977	0.323	1.344	0.748-2.416
*GNAS1* genotype	7.422	0.006	0.367	0.178-0.755
Line	0.01	0.919	0.970	0.54-1.744

## 3 讨论

小分子TKI是晚期NSCLC 21世纪最重要的治疗进展，肿瘤组织*EGFR*突变是最重要的疗效预测因子^[1]^，中国的OPTIMAL^[10]^、日本的WJTOG3405^[11]^和NEJ002^[12]^及欧洲EURTAC^[13]^等多项Ⅲ期随机对照研究结果显示*EGFR*突变的晚期NSCLC，接受TKI治疗的有效率可以高达58%-82%，明显高于传统的第3代化疗新药与铂的两药联合治疗，PFS也较化疗组明显延长。

然而，临床实践过程中，多数的晚期NSCLC患者因肿瘤组织采集困难、患者依从性差等多种原因导致无法取得足够的肿瘤组织量进行*EGFR*突变状态检测，临床上能够获得基因突变检测标本的患者仅约20%（来自ISEL研究的数据）^[2]^。

对这些*EGFR*突变状态未明的患者，疗效预测目前主要依赖一些临床因素，如亚裔、女性、不吸烟、腺癌等^[14]^。如何对这些患者进行治疗前的疗效预估是我们临床实践中面临的难题。

多项基础研究发现，TKI诱导肿瘤细胞凋亡可能是其重要的作用机制之一，研究发现凋亡相关基因，如*BIM*基因^[15, 16]^、*Bcl*-2基因^[17]^参与TKI诱导的细胞凋亡。不论吉非替尼还是厄洛替尼都可在肿瘤细胞内通过Bcl-2/Bcl-xL复合体来调节下游的IP3R3蛋白导致肿瘤细胞产生凋亡抵抗^[18]^，敲除*BCL*-2基因可以增加肿瘤细胞对TKI的敏感性^[17]^。

*GNAS1*基因位于20号染色体，包含13个外显子，其中T393C多态性与肿瘤组织Gs蛋白α亚单位mRNA表达增加相关^[7]^。而且，Gs蛋白α亚单位表达增加会促进凋亡发生^[8]^。

本研究所有样本共116例，其中*GNAS1*基因CC患者有13例，ORR为30.8%，但DCR为46.2%；TT型患者46例，CT型患者57例，ORR分别为23.9%和33.3%，而DCR分别为65.2%和80.7%。在疾病控制率方面，CC型对比*GNAS1*的其它基因型更低，提示预后差（DCR: 46.2% *vs* 73.8%, *P*=0.039），女性、不吸烟、腺癌患者是TKI治疗的优势人群。单因素分析中位PFS，女性长于男性（10.2个月*vs* 4.6个月，χ^2^ =4.24，*P*=0.04）；不吸烟者长于有吸烟史者（11.9个月*vs* 2.5个月，χ^2^ =15.28，*P* < 0.001）；病理类型为腺癌长于其他类型（11.9个月*vs* 4.1个月，χ^2^ =13.06，*P* < 0.001），差异均有统计学意义。研究还发现*GNAS1*基因型为CC型者预后差，中位PFS短于其它基因型的患者（2.3个月*vs* 6.0个月，*P*=0.005）。多因素分析结果也证实*GNAS1*基因T393C基因多态性为PFS的独立预后因素。其他研究^[19]^也发现，CC型肿瘤患者的易于复发，我们的既往研究发现*GNAS1*基因T393C多态性TT型与吉西他滨为基础的较好的化疗疗效相关^[20]^。

因此，本研究结果进一步从临床中证实了*GNAS1*基因T393C多态性能够作为预测*EGFR*突变未明NSCLC患者TKI治疗的疗效的指标之一。我们推测*GNAS1*基因型为CC的患者，由于影响了TKI诱导细胞凋亡通路，导致近期疗效欠佳，具体的机制值得进一步研究。本研究还发现不同*GNAS1*基因型患者间的近期有效率并无统计学意义。在*EGFR*突变未明的中国患者，*GNAS1* T393C基因型为CC占比为11.2%，因不同人群和不同种族可能会存在基因突变频率的差异。

本研究为回顾性研究，且样本量有限，有待后续更深入研究及期待前瞻性多中心的大型临床研究为肺癌的治疗提供更高级别的循证医学证据。
